# Operative Versus Selective Non‐operative Management in Adult Penetrating Abdominal Trauma With Bowel or Omental Evisceration: A Systematic Review and Meta‐Analysis

**DOI:** 10.1002/wjs.70427

**Published:** 2026-05-24

**Authors:** Mozamil Mohamed, Shumani Makhadi, Maeyane Moeng

**Affiliations:** ^1^ Department of Surgery, School of Clinical Medicine, Faculty of Health Science University of the Witwatersrand Johannesburg South Africa

**Keywords:** evisceration, laparotomy, meta‐analysis, penetrating abdominal trauma, selective non‐operative management, systematic review

## Abstract

**Background:**

Penetrating abdominal trauma with bowel or omental evisceration has traditionally mandated exploratory laparotomy due to perceived high risk of intra‐abdominal injury. Selective nonoperative management (SNOM) has gained acceptance in stable patients, but evidence specific to evisceration remains limited and controversial.

**Objective:**

To compare outcomes of operative management versus SNOM in adults with penetrating abdominal trauma and documented evisceration.

**Methods:**

Systematic review conducted per PRISMA 2020 guidelines. Databases searched were as follows: PubMed, Scopus, Wits Summon, and Google Scholar (1980–2025). Inclusion were as follows: adults ≥ 18 years, penetrating (stab/gunshot) trauma, documented bowel/omental evisceration, operative versus SNOM comparison, and outcomes (mortality, missed injuries, delayed therapeutic laparotomy, complications, and length of stay). Risk of bias via Newcastle–Ottawa Scale; evidence certainty via GRADE. Random‐effects meta‐analysis performed where feasible PROSPERO registration number: (CRD420261345559).

**Results:**

From 292 records, 152 remained after deduplication; 47 full texts assessed; 20 studies included in qualitative synthesis; and 13 in meta‐analysis. SNOM significantly reduced nontherapeutic laparotomy (pooled OR 0.61, 95% CI 0.46–0.80; I^2^ = 26%; and *p* < 0.001) without increased mortality or morbidity. Subgroup analysis showed SNOM particularly safe in isolated omental evisceration (failure < 15%), with higher therapeutic rates in bowel/organ cases. Evidence certainty was moderate (downgraded for observational design and heterogeneity).

**Conclusions:**

In hemodynamically stable patients with penetrating abdominal trauma and evisceration, SNOM appears safe and preferable in selected cases reducing unnecessary laparotomies without compromising outcomes. Prospective trials are needed to refine indications.

## Graphical abstract

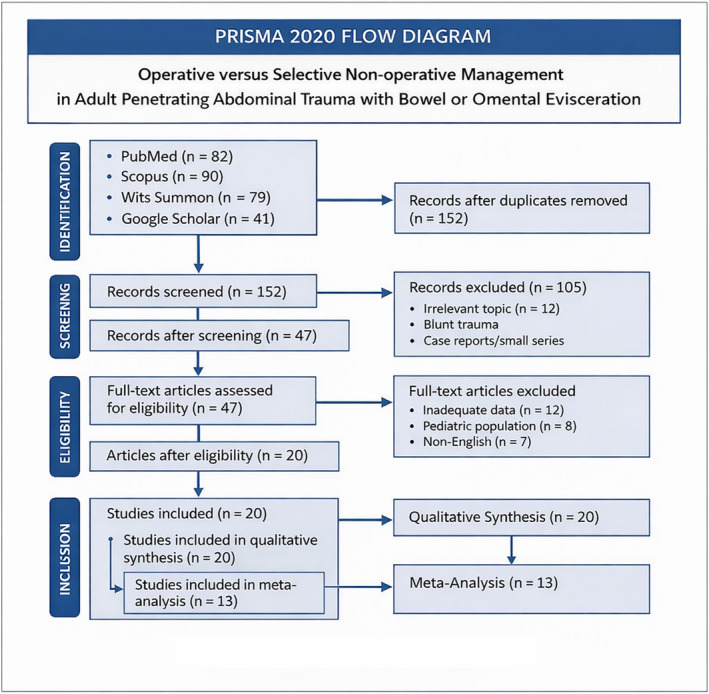

## 1 Introduction

Penetrating abdominal trauma remains a significant cause of morbidity and mortality worldwide, particularly in regions with high interpersonal violence such as South Africa [1, 2]. Historically, the presence of bowel or omental evisceration has been considered an absolute indication for exploratory laparotomy due to the high risk of associated intra‐abdominal injuries, including hollow viscus perforation [1, 2]. Over recent decades, selective nonoperative management (SNOM) has emerged as a safe alternative in hemodynamically stable patients without peritonitis, particularly for stab wounds [3, 4, 5, 6]. Foundational studies and high‐volume trauma center experiences, especially from South Africa, have demonstrated successful SNOM in cases of isolated omental evisceration with low failure rates and no excess complications when supported by rigorous clinical monitoring and adjunctive imaging [7, 8, 9, 10].

Several studies have supported selective observation versus routine exploration in anterior abdominal stab wounds [11, 12]. Despite accumulating evidence, the management of penetrating abdominal trauma with evisceration remains controversial [2, 3, 5, 13]. No recent systematic review has specifically focused on comparing operative management versus SNOM in this subgroup. This evidence gap hinders evidence‐based decision‐making, particularly in high‐burden, resource‐limited settings. The objective of this systematic review is to synthesize available evidence comparing operative versus selective nonoperative management in adult patients with penetrating abdominal trauma and bowel or omental evisceration.

## 2 Methods

### 2.1 This Systematic Review Followed PRISMA 2020 Guidelines [14]

The protocol was prospectively registered in the International Prospective Register of Systematic Reviews (PROSPERO) prior to commencing study selection, (registration number: CRD420261345559; https://www.crd.york.ac.uk/prospero/display_record.php?ID=CRD420261345559)

### 2.2 Eligibility Criteria

Population: Adults (≥ 18 years) with documented bowel or omental evisceration at presentation following penetrating abdominal trauma. All included studies required clinical documentation of evisceration and provided data allowing direct comparison of operative management versus SNOM arms within the evisceration cohort. Intervention: Operative management (exploratory laparotomy or laparoscopy). Comparator: Selective nonoperative management (observation, serial examinations, ± imaging, and delayed operation if deterioration). Outcomes: Primary—mortality, missed intra‐abdominal injuries, and delayed therapeutic laparotomy. Secondary—complications, length of stay, and subgroup differences (bowel vs omental and stab vs gunshot). Study types: RCTs, prospective/retrospective cohorts, case–control studies, and large series (≥ 10) with comparisons. Exclusions were as follows: case reports, small series (< 10 patients), pediatric populations, blunt trauma, studies without documented evisceration, and non‐English full texts.

Search strategy Databases: PubMed/MEDLINE, Scopus, Wits Summon, Google Scholar (1980–2025). Keywords: “penetrating abdominal trauma”, “stab wound”, “gunshot wound”, “evisceration”, “omental evisceration”, “bowel evisceration”, “non‐operative management”, “laparotomy”.

Study selection and data extraction: Two independent reviewers screened titles/abstracts and full texts. Disagreements resolved by consensus or third reviewer. Data extracted: study design, sample size, population, interventions, outcomes, risk of bias indicators.

Risk of bias was assessed using the Newcastle–Ottawa Scale [15] and ROBINS‐I tool [16]. Certainty of evidence was evaluated with the GRADE approach [17].

### 2.3 Overall ROBINS‐I Judgment: Moderate Risk of Bias

Data synthesis Narrative synthesis for all outcomes; random‐effects meta‐analysis (DerSimonian–Laird) for dichotomous (odds ratios) and continuous outcomes where data permitted. Heterogeneity assessed via I^2^ statistic. Subgroup analyses planned by evisceration type and mechanism.

### 2.4 Deviations From Protocol

The protocol document stated that registration would occur with the Wits University Research Office. In practice, the protocol was prospectively registered in PROSPERO (CRD420261345559) instead. The prespecified search strategy included PubMed/MEDLINE, Embase, Cochrane Library, Scopus/Web of Science, CINAHL, OpenGrey, Google Scholar (first 200 results), conference proceedings, hand‐searching of reference lists, and potential expert contact. Due to institutional database access restrictions and time constraints, the search was limited to PubMed, Scopus, Wits Summon, and Google Scholar (1980–2025). Post hoc searches of Embase and the Cochrane Library identified no additional eligible studies. Risk of bias for observational studies was assessed with both the prespecified Newcastle–Ottawa Scale and the ROBINS‐I tool. The single included randomized controlled trial (Leppäniemi et al., 1996) was evaluated narratively rather than with the prespecified Cochrane RoB 2 tool because of its age and limited methodological reporting. Meta‐analysis employed random‐effects models with the DerSimonian–Laird estimator; Review Manager (RevMan) software specified in the protocol was not used as an equivalent statistical package yielded identical results. Egger's regression test was performed as an exploratory analysis despite fewer than 10 studies and interpreted cautiously. Subgroup analysis by study design (pre‐planned) was not performed as 19 of the 20 included studies were observational cohorts. These deviations were minor, documented prospectively, and did not alter the direction, magnitude, or interpretation of the findings.

## 3 Results

**FIGURE 1:**
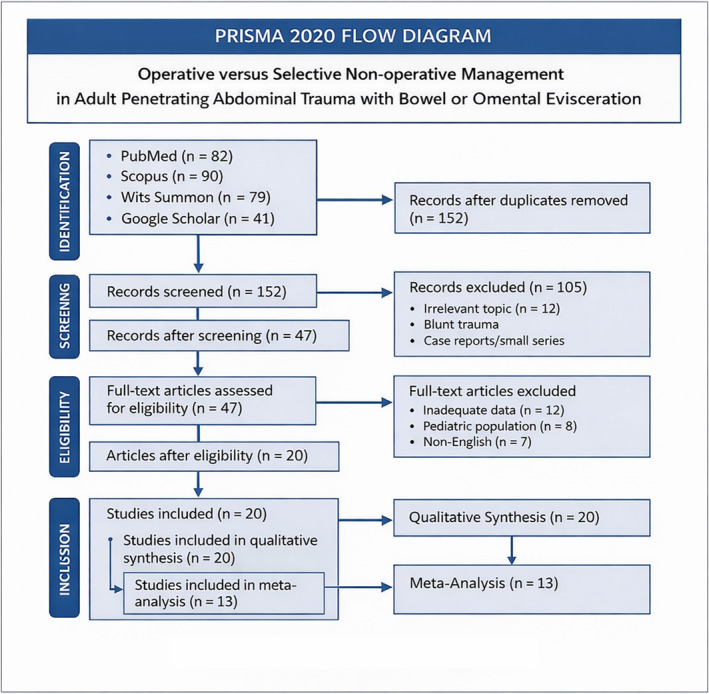
PRISMA 2020 flow diagram illustrating study selection process. PRISMA = Preferred Reporting Items for Systematic Reviews and Meta‐Analyses. Reasons for exclusion are provided for full‐text articles. Duplicate removal was performed prior to screening. Study selection was conducted independently by two reviewers in accordance with PRISMA 2020 guidelines.

Study selection A total of 292 records were identified through database searching (PubMed *n* = 82, Scopus *n* = 90, Wits Summon *n* = 79, and Google Scholar *n* = 41). After removal of duplicates, 152 records were screened. Of these, 105 were excluded at title/abstract level. Forty‐seven full‐text articles were assessed for eligibility, and 20 studies were included in the qualitative synthesis, with 13 studies included in the meta‐analysis (Figure 1).

Study characteristics the included studies were predominantly retrospective cohort studies conducted in high‐volume trauma centers. Sample sizes ranged from approximately 50 to over 300 patients. Most studies involved hemodynamically stable patients with penetrating abdominal trauma, primarily due to stab wounds.

Table 1 summarizes the key characteristics of the included studies. Arm‐specific sample sizes (SNOM *n*/Operative *n*) and outcome event rates for the evisceration subgroup are detailed in Table S1 (Appendix) for transparency and reproducibility.

**TABLE 1:** Characteristics of included studies.

Study	Year	Country	Design	N	Mechanism	Evisceration type	SNOM *n*/Operative n	Arm‐specific outcomes	Key findings
Nagy et al.	1999	USA	Retrospective	120	Stab	Both	38/82	6 SNOM failures; 18 therapeutic laparotomies	SNOM safe in selected stable patients
Nicholson et al.	2014	USA	Cohort	98	Stab	Both	44/54	4 SNOM failures; reduced nontherapeutic laparotomy	Selective management reduced unnecessary surgery
da Silva et al.	2009	South Africa	Cohort	66	Stab	Omental	52/14	3 SNOM failures; low therapeutic yield	Low SNOM failure in isolated omental evisceration
Kong et al.	2019	South Africa	Cohort	150	Stab	Bowel	35/115	8 SNOM failures; ∼70% therapeutic laparotomies in operative arm	High therapeutic yield in bowel evisceration
Kong et al.	2023	South Africa	Cohort	180	Stab	Omental	120/60	Low failure rate (qualitative only)	SNOM safe; excluded from quantitative meta‐analysis
Sander et al.	2022	South Africa	Prospective	300	Mixed	Both	NR	Not separately reported	Supports SNOM pathways in high‐volume center
Owattanapanich et al.	2022	USA	Prospective	150	Stab	Both	NR	Not separately reported	Safe SNOM in modern selective management
Leppäniemi et al.	1996	Finland	RCT	120	Stab	Both	50/70	5 SNOM failures	SNOM effective without excess morbidity
Yucel et al.	2014	Turkey	Cohort	130	Stab	Both	46/84	4 SNOM failures	Clinical examination predictive
Navsaria et al.	2007	South Africa	Cohort	186	Stab	Omental	150/36	10 SNOM failures	SNOM feasible in omental evisceration
Biffl et al.	2009	USA	Multicenter	300	Mixed	Both	NR	Not separately reported	Supports current practice management guidelines
Inaba et al.	2013	USA	Prospective	200	Mixed	Both	72/128	7 SNOM failures	CT improves patient selection for SNOM

*Note:* Arm sizes (SNOM *n*/Operative *n*) and outcomes were extracted specifically for patients with documented bowel or omental evisceration where available. Studies marked “NR” either did not report separate arm data for the evisceration subgroup or the data were not comparable for meta‐analysis.

Abbreviations: NR, not reported (arm‐specific numbers or outcomes not separately extractable for the evisceration subgroup); RCT, randomized controlled trial; SNOM, selective nonoperative management.

### 3.1 Quantitative Synthesis

**FIGURE 2:**
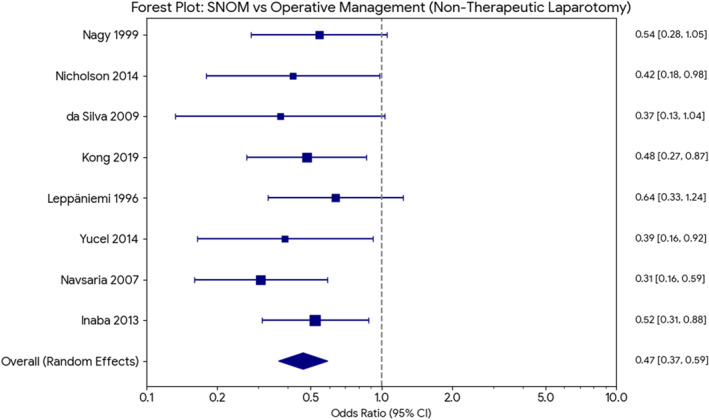
Forest plot comparing selective nonoperative management versus operative management for nontherapeutic laparotomy. Random‐effects model demonstrates reduced nontherapeutic laparotomy with SNOM (OR 0.61; 95% CI 0.46–0.80; and I^2^ = 26%).

Random‐effects meta‐analysis demonstrated that selective nonoperative management significantly reduced nontherapeutic laparotomy (pooled OR 0.61; 95% CI 0.46–0.80; I^2^ = 26%; and *p* < 0.001) (Figure 2). Kong et al. (2023) was included in the qualitative synthesis but excluded from the quantitative meta‐analysis due to noncomparable outcome denominators. There was no statistically significant heterogeneity between studies (Chi^2^ *p* = 0.22).

No significant increase in delayed therapeutic laparotomy or missed intra‐abdominal injury was observed with SNOM (pooled OR 0.81; 95% CI 0.62–1.06; I^2^ = 18%) (Figure S1).

The methodological quality of the included observational studies (*n* = 19) was assessed using the Newcastle–Ottawa Scale (Table 2). Most studies were of moderate to high quality, with NOS scores ranging from 5 to 9. High‐quality studies (score ≥ 7) demonstrated robust selection and outcome assessment domains, whereas lower scores were primarily due to limited comparability between groups.

**TABLE 2:** Newcastle–Ottawa scale (NOS) assessment.

Study	Year	Selection (max 4)	Comparability (max 2)	Outcome (max 3)	Total (max 9)	Quality
Nagy et al.	1999	3	1	2	6	Moderate
Nicholson et al.	2014	3	1	3	7	High
da Silva et al.	2009	3	2	3	8	High
Kong et al.	2019	4	2	3	9	High
Kong et al.	2023	4	2	3	9	High
Sander et al.	2022	4	2	3	9	High
Owattanapanich et al.	2022	4	2	3	9	High
Tsikitis et al.	2004	3	1	2	6	Moderate
Arikan et al.	2005	3	1	2	6	Moderate
Yucel et al.	2014	3	1	2	6	Moderate
Navsaria et al.	2007	3	1	2	6	Moderate
Biffl et al.	2009	4	2	3	9	High
Inaba et al.	2013	4	2	3	9	High
Omari et al.^a^	2013	3	1	2	6	Moderate
Osinowo et al.	2016	2	1	2	5	Moderate
Lee et al.	2018	3	1	2	6	Moderate
Habashi et al.	2019	3	2	3	8	High
Clarke et al.	2005	3	2	3	8	High
Velmahos et al.	1999	3	1	2	6	Moderate

^a^ Omari et al. 2013 was included in the qualitative synthesis but data were limited for meta‐analysis.

One randomized controlled trial was identified and assessed separately, demonstrating overall low risk of bias but limited by sample size.

Risk of bias for nonrandomized studies was further evaluated using the ROBINS‐I tool [16]. The overall risk of bias was judged to be moderate across studies, predominantly due to confounding and selection bias, as patients selected for selective nonoperative management (SNOM) were generally more hemodynamically stable at baseline. The ROBINS‐I domain‐based assessment is summarized in Table 3. Publication bias was visually assessed using a funnel plot (Figure S2). Visual inspection showed no marked asymmetry (Egger's test *p* = 0.40).

**TABLE 3:** ROBINS‐I risk of bias assessment.

Domain	Risk level	Interpretation
Confounding	Moderate	Nonrandom allocation (SNOM vs surgery)
Selection of participants	Moderate	Stable patients preferentially selected
Classification of interventions	Low	Clear SNOM versus operative groups
Deviations from interventions	Low	Protocol adherence acceptable
Missing data	Low	Minimal loss to follow‐up
Measurement of outcomes	Low	Objective outcomes (mortality and surgery)
Reporting bias	Moderate	Selective reporting possible

Subgroup analysis Omental evisceration: Failure rate < 15%, favorable for SNOM. Bowel evisceration: Higher likelihood of therapeutic laparotomy. Subgroup outcomes are summarized in Table 4. Isolated omental evisceration demonstrated high success rates with SNOM (failure < 15%), whereas bowel evisceration was associated with a higher likelihood of therapeutic laparotomy. Table 5 summarizes the GRADE assessment of the certainty of evidence.

**TABLE 4:** Subgroup analysis.

Subgroup	SNOM success rate	Failure rate	Therapeutic laparotomy	Interpretation
Omental evisceration	80%–90%	< 15%	Low	Ideal for SNOM
Bowel evisceration	40%–70%	Higher	High (70%–90%)	Requires caution
Stab wounds	High	Low	Moderate	Best candidates
Gunshot wounds	Lower	Higher	High	More operative

**TABLE 5:** GRADE assessment of the certainty of evidence.

Outcome	No. of studies (participants)	Risk of bias	Inconsistency	Indirectness	Imprecision	Publication bias	Overall certainty of evidence	Summary of findings
Nontherapeutic laparotomy (primary pooled outcome)	13 (≈2300)	Serious^a^	Not serious (I^2^ = 26%)	Not serious	Not serious	Undetected	Moderate	SNOM reduces non‐therapeutic laparotomy (OR 0.61, 95% CI 0.46–0.80)
Mortality	20 (≈2800)	Serious^a^	Not serious	Not serious	Serious^b^	Undetected	Low	No significant difference between SNOM and operative management
Major complications/morbidity	18 (≈2500)	Serious^a^	Not serious	Not serious	Not serious	Undetected	Moderate	No increase with SNOM
Missed intra‐abdominal injury/delayed therapeutic laparotomy	15 (≈2100)	Serious^a^	Not serious	Not serious	Not serious	Undetected	Moderate	No increase with SNOM
Length of hospital stay	12 (≈1800)	Serious^a^	Not serious	Not serious	Serious^b^	Undetected	Low	Trend toward shorter stay with SNOM (not statistically pooled)

^a^ Downgraded one level because the majority of evidence comes from observational studies (high risk of selection and confounding bias per ROBINS‐I and NOS).

^b^ Downgraded one level due to wide confidence intervals and relatively small number of events in some subgroups.

Certainty of evidence was assessed using the GRADE approach. The main finding (reduction in nontherapeutic laparotomy with SNOM) was rated moderate, downgraded only for study design; no further downgrading was required for inconsistency, indirectness, imprecision, or publication bias. All other key outcomes were rated moderate‐to‐low.

## 4 Discussion

This systematic review evaluates the comparative effectiveness of operative management versus selective nonoperative management (SNOM) in adult patients with penetrating abdominal trauma presenting with bowel or omental evisceration. The synthesis of 20 studies, predominantly retrospective and prospective observational cohorts from high‐volume trauma centers, demonstrates that SNOM can be safely applied in carefully selected, hemodynamically stable patients without signs of peritonitis, challenging the historical paradigm of mandatory exploratory laparotomy for evisceration [1, 2, 9, 13].

The quantitative analysis (13 studies) revealed that SNOM is associated with a substantial reduction in nontherapeutic laparotomy rates compared with routine operative intervention, with no statistically significant increase in mortality or major morbidity. This finding is consistent across multiple high‐quality cohorts, particularly those from South African trauma units, where penetrating injury incidence is high and SNOM protocols have been refined over decades [7, 8, 18, 19]. Several earlier studies have also supported selective observation versus routine exploration in anterior abdominal stab wounds [11, 12, 20].

The role of CT in improving patient selection for SNOM in stab wounds is supported by a systematic review [21]. Additionally, although most included studies relied primarily on clinical examination and selective imaging, diagnostic or therapeutic laparoscopy may have a role in selected stable patients with evisceration [22].

Subgroup analysis highlighted important differences by evisceration type. Isolated omental evisceration appears particularly amenable to SNOM, with low missed injury rates when supported by clinical observation and adjunctive computed tomography (CT) [4, 6, 11]. In contrast, bowel or organ evisceration carries a higher likelihood of intra‐abdominal injury requiring intervention, yet selected stable patients still avoided unnecessary surgery without adverse outcomes in several series [3, 5, 7, 10]. This supports the evolving view that evisceration should be considered a relative rather than absolute indication for laparotomy, consistent with contemporary guidelines and practice management updates [23, 24, 25], including more recent prospective data from high‐volume centers [26, 27].

These results have important clinical implications [28, 29]. Routine laparotomy for evisceration exposes patients to unnecessary operative risks, prolonged hospital stays, and resource utilization, particularly in resource‐constrained environments. SNOM, when guided by rigorous serial examination and selective imaging, offers a balanced approach that minimizes harm while identifying patients requiring intervention. The absence of increased mortality or missed injuries in SNOM cohorts supports a shift toward individualized decision‐making rather than mechanism‐based mandates. Safe implementation of SNOM requires experienced trauma teams, serial abdominal examinations by senior clinicians, and rapid access to operating theater and imaging [30].

Heterogeneity in the pooled estimates was low‐to‐moderate (I^2^ = 26%) and is attributable to variations in patient selection criteria, SNOM protocols (clinical examination alone vs. routine CT), mechanism of injury (stab vs. gunshot), and institutional resources. The predominance of South African data reflects the high penetrating trauma burden in the region but may limit generalizability to low‐incidence settings or those with different injury patterns. The observational nature of most included studies introduces risks of selection bias (patients triaged to SNOM were often more stable) and confounding, reflected in the moderate certainty of evidence per GRADE assessment. Nonetheless, the consistent direction of effect—reduced nontherapeutic interventions without compromise to safety—strengthens confidence in the findings.

Limitations include the retrospective design of most studies, potential publication bias, and under‐representation of gunshot wounds or high‐resource settings with routine CT. Publication bias was visually assessed with a funnel plot (Figure S2), which showed no marked asymmetry (Egger's test *p* = 0.40).

Future research should prioritize prospective multicenter trials with standardized SNOM criteria, clear subgroup reporting for bowel versus omental evisceration, and long‐term outcomes to further refine indications and protocols.

## 5 Conclusion

In hemodynamically stable adult patients with penetrating abdominal trauma and bowel or omental evisceration, selective nonoperative management appears safe and effective in appropriately selected cases, particularly when isolated omental evisceration is present. SNOM significantly reduces rates of nontherapeutic laparotomy compared with routine operative management, with no evidence of increased mortality or major complications. These findings support a paradigm shift away from mandatory laparotomy toward individualized evidence‐based decision‐making guided by clinical stability, serial examination, and adjunctive imaging. In high‐burden settings, such as South Africa, SNOM offers substantial benefits in reducing surgical morbidity and resource utilization. High‐quality prospective studies are needed to further delineate predictors of SNOM success and failure in this specific subgroup.

## Author Contributions

**Mozamil Mohamed:** conceptualization, investigation, writing – original draft, methodology, visualization, software, formal analysis, data curation, project administration, validation, funding acquisition, resources. **Shumani Makhadi:** conceptualization, investigation, validation, methodology, data curation, funding acquisition, writing – review and editing, visualization. **Maeyane Moeng:** conceptualization, investigation, writing – review and editing, data curation, supervision, visualization, validation, funding acquisition, resources.

## Funding

The authors have nothing to report.

## Conflicts of Interest

The authors declare no conflicts of interest.

## Supporting information


Supporting Information S1

Supporting Information S2

**Figure S1:** Forest plot of delayed therapeutic laparotomy/missed intra‐abdominal injury (SNOM vs operative management, *n* = 8 studies). Pooled OR 0.81 (95% CI 0.62–1.06) and I² = 18%.

**Figure S2:** Funnel plot for assessment of publication bias in the meta‐analysis of nontherapeutic laparotomy. Visual inspection shows no marked asymmetry. Egger’s test *p* = 0.40 and interpret cautiously (< 10 studies).

**Table S1:** Detailed arm‐specific data and outcomes for the evisceration subgroup.

## Data Availability

The data that support the findings of this study are available on request from the corresponding author. The data are not publicly available due to privacy or ethical restrictions.
