# Supplementary material for: Operative Versus Selective Non‐operative Management in Adult Penetrating Abdominal Trauma With Bowel or Omental Evisceration: A Systematic Review and Meta‐Analysis
Source: World J Surg. 2026 May 24;50(7):2008–16. doi: 10.1002/wjs.70427 (PMC13356563; doi:10.1002/wjs.70427)
Supplement: Supplementary file 1 — Supporting Information S1 [file WJS-50-2008-s002.docx]

**Appendix: Full Electronic Search Strategy**

The full electronic search strategy for PubMed is presented in the Appendix. Similar strategies were adapted for other databases (Scopus, Wits Summon, and Google Scholar

**Database:** PubMed/MEDLINE (searched from 1 January 1980 to 31 December 2025)

**Search Strategy:**

("abdominal injuries"[MeSH Terms] OR "abdominal trauma" OR "abdominal stab" OR "abdominal gunshot" OR "penetrating abdominal" OR "abdominal penetrating trauma" OR "stab wound abdomen" OR "gunshot wound abdomen")

AND

(evisceration[Title/Abstract] OR "omental evisceration"[Title/Abstract] OR "bowel evisceration"[Title/Abstract] OR "viscus evisceration"[Title/Abstract] OR "intestinal evisceration"[Title/Abstract] OR "omentum evisceration"[Title/Abstract] OR eviscerat*[Title/Abstract])

AND

("non-operative management"[Title/Abstract] OR "nonoperative management"[Title/Abstract] OR "selective non-operative"[Title/Abstract] OR "selective nonoperative"[Title/Abstract] OR SNOM[Title/Abstract] OR "conservative management"[Title/Abstract] OR "non-operative treatment"[Title/Abstract] OR observation[Title/Abstract] OR "watchful waiting"[Title/Abstract] OR "expectant management"[Title/Abstract])

OR

(laparotomy[Title/Abstract] OR "exploratory laparotomy"[Title/Abstract] OR "operative management"[Title/Abstract] OR surgery[Title/Abstract] OR "surgical management"[Title/Abstract])

AND

("adult"[MeSH Terms] OR adult*[Title/Abstract])

**Limits applied:**

- Publication date: 1980/01/01 to 2025/12/31
- Language: English (as per eligibility criteria)

**Total records retrieved from PubMed:** 82
