# Supplementary material for: Operative Versus Selective Non‐operative Management in Adult Penetrating Abdominal Trauma With Bowel or Omental Evisceration: A Systematic Review and Meta‐Analysis
Source: World J Surg. 2026 May 24;50(7):2008–16. doi: 10.1002/wjs.70427 (PMC13356563; doi:10.1002/wjs.70427)
Supplement: Supplementary file 2 — Supporting Information S2 [file WJS-50-2008-s003.docx]

Supplementary Figure S1. Forest plot of delayed therapeutic laparotomy / missed intra-abdominal injury (SNOM vs operative management, n = 8 studies). Pooled OR 0.81 (95% CI 0.62–1.06); I² = 18%.

Supplementary Figure S2. Funnel plot for assessment of publication bias in the meta-analysis of non-therapeutic laparotomy. Visual inspection shows no marked asymmetry. Egger’s test p = 0.40; interpret cautiously (<10 studies).
