# Supplementary figures and images for: Operative Versus Selective Non‐operative Management in Adult Penetrating Abdominal Trauma With Bowel or Omental Evisceration: A Systematic Review and Meta‐Analysis
Source: World J Surg. 2026 May 24;50(7):2008–16. doi: 10.1002/wjs.70427 (PMC13356563; doi:10.1002/wjs.70427)

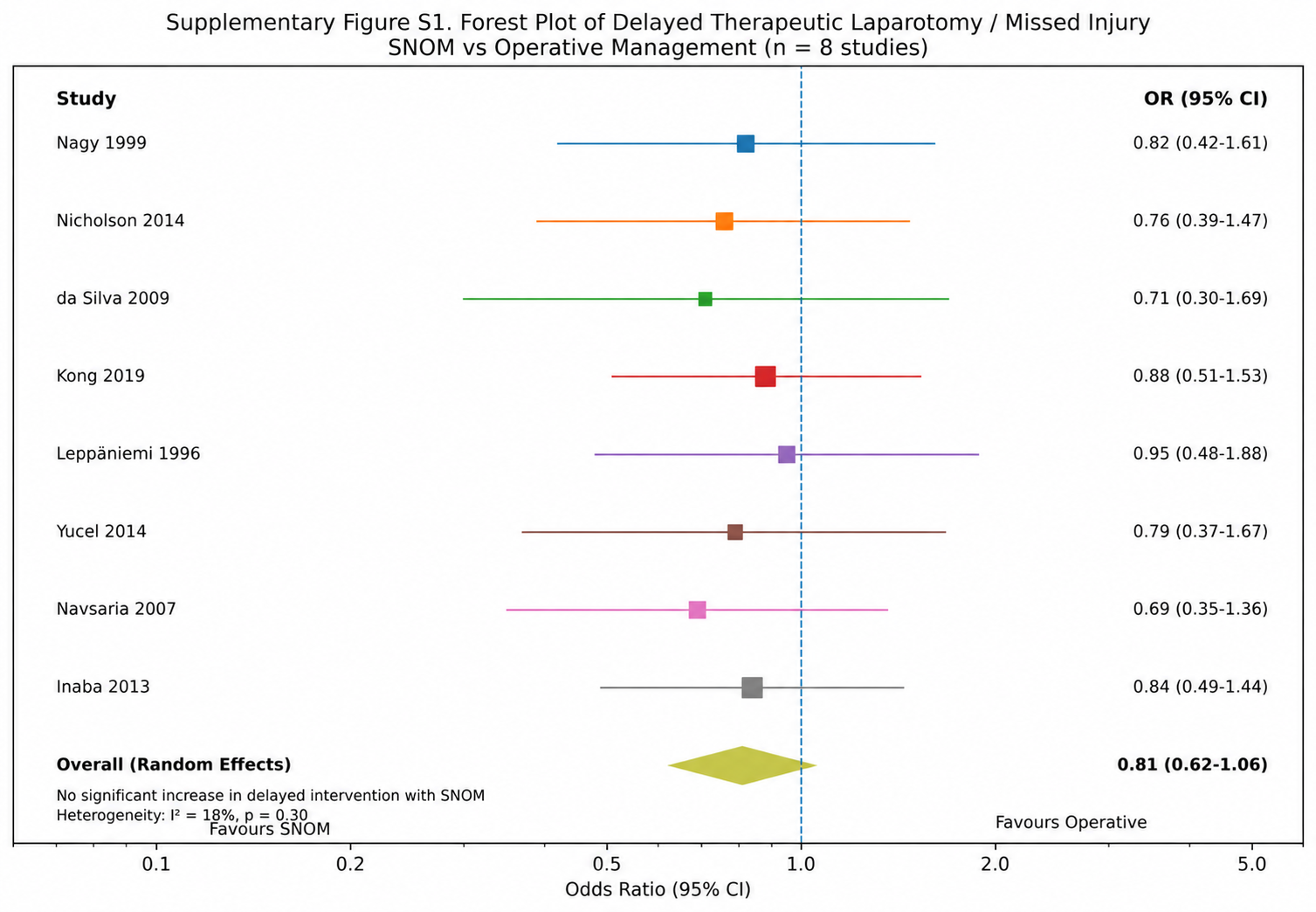

Supplement: Supplementary file 3 — Figure S1: Forest plot of delayed therapeutic laparotomy/missed intra‐abdominal injury (SNOM vs operative management, n = 8 studies). Pooled OR 0.81 (95% CI 0.62–1.06) and I² = 18%. [file WJS-50-2008-s005.png]

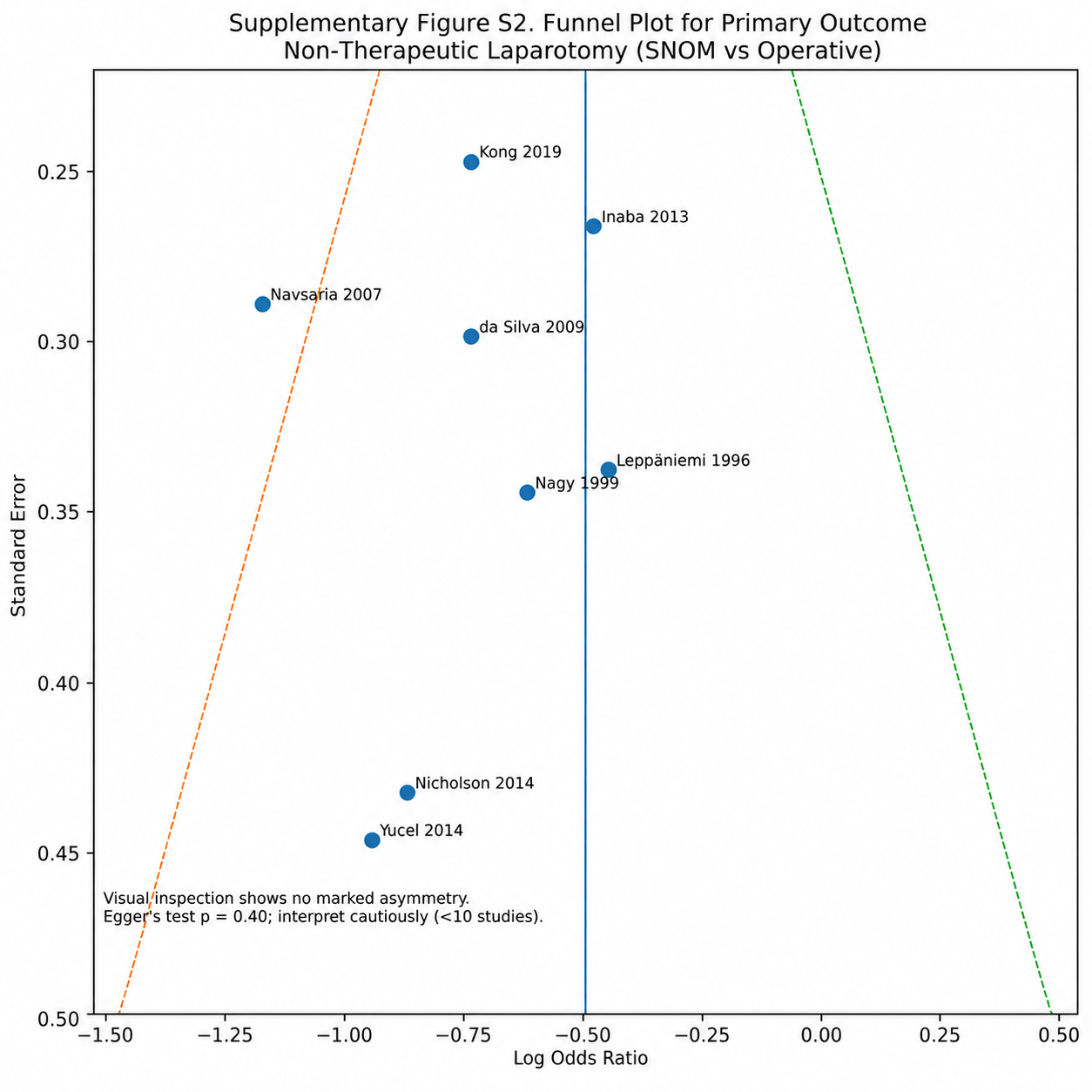

Supplement: Supplementary file 4 — Figure S2: Funnel plot for assessment of publication bias in the meta‐analysis of nontherapeutic laparotomy. Visual inspection shows no marked asymmetry. Egger’s test p = 0.40 and interpret cautiously (< 10 studies). [file WJS-50-2008-s004.png]
