# Supplementary material for: Operative Versus Selective Non‐operative Management in Adult Penetrating Abdominal Trauma With Bowel or Omental Evisceration: A Systematic Review and Meta‐Analysis
Source: World J Surg. 2026 May 24;50(7):2008–16. doi: 10.1002/wjs.70427 (PMC13356563; doi:10.1002/wjs.70427)
Supplement: Supplementary file 5 — Table S1: Detailed arm‐specific data and outcomes for the evisceration subgroup. [file WJS-50-2008-s001.docx]

**Supplementary Table S1.** Detailed arm-specific data and outcomes for the evisceration subgroup.

| **Study** | **Year** | **Total N** | **SNOM n** | **Operative n** | **SNOM Failures (Delayed Therapeutic Laparotomy)** | **Non-Therapeutic Laparotomies in Operative Arm** | **Therapeutic Laparotomies in Operative Arm** | **Major Complications (SNOM vs Operative)** | **Mortality (SNOM vs Operative)** | **Notes / Key Comments** |
| --- | --- | --- | --- | --- | --- | --- | --- | --- | --- | --- |
| Nagy et al. | 1999 | 120 | 38 | 82 | 6 | 64 | 18 | NR | NR | SNOM safe in stable patients |
| Nicholson et al. | 2014 | 98 | 44 | 54 | 4 | Reduced (exact NR) | NR | No significant difference | No difference | Selective management effective |
| da Silva et al. | 2009 | 66 | 52 | 14 | 3 | Low yield | Low | Low in SNOM | 0 vs 0 | Focused on omental evisceration |
| Kong et al. | 2019 | 150 | 35 | 115 | 8 | NR | ~70–80% | Comparable | Low | High therapeutic yield in bowel evisceration |
| Kong et al. | 2023 | 180 | 120 | 60 | Low (<15%) | NR | NR | No increase with SNOM | No difference | Excluded from meta-analysis due to non-comparable denominators |
| Leppäniemi et al. | 1996 | 120 | 50 | 70 | 5 | High | NR | No excess morbidity | No difference | Only RCT included |
| Yucel et al. | 2014 | 130 | 46 | 84 | 4 | NR | NR | Clinical exam reliable | NR | Predictive value of examination |
| Navsaria et al. | 2007 | 186 | 150 | 36 | 10 | High | NR | Feasible with low complications | Low | Large omental SNOM series |
| Inaba et al. | 2013 | 200 | 72 | 128 | 7 | Improved with CT | NR | CT aids selection | No difference | Role of CT emphasised |
| Sander et al. | 2022 | 300 | NR | NR | Low | NR | NR | Supports SNOM | Low | High-volume prospective experience |
| Owattanapanich et al. | 2022 | 150 | NR | NR | Low | NR | NR | Safe SNOM | Low | Modern US experience |
| Biffl et al. | 2009 | 300 | NR | NR | NR | NR | NR | Supports guidelines | NR | Multicentre study |

**Abbreviations:** SNOM = selective non-operative management; NR = not reported or not extractable for the evisceration-specific subgroup; RCT = randomised controlled trial.

**Footnote:** This supplementary table provides more detailed arm-specific data extracted for the subgroup of patients with documented bowel or omental evisceration. Data were obtained from the original publications where available. Studies with “NR” either did not report separate outcomes for the evisceration cohort or the data were not suitable for pooled quantitative analysis. Failure rates in SNOM arms generally remained low (<15%) in omental evisceration cases.
